# MAXENT3D_PID: An Estimator for the Maximum-Entropy Trivariate Partial Information Decomposition

**DOI:** 10.3390/e21090862

**Published:** 2019-09-03

**Authors:** Abdullah Makkeh, Daniel Chicharro, Dirk Oliver Theis, Raul Vicente

**Affiliations:** 1 Institute of Computer Science, University of Tartu, 51014 Tartu, Estonia; 2 Neural Computation Laboratory, Center for Neuroscience and Cognitive Systems@UniTn, Istituto Italiano di Tecnologia, 38068 Rovereto (TN), Italy

**Keywords:** multivariate information theory, partial information decomposition, cone programming, synergy, redundancy, Python

## Abstract

Partial information decomposition (PID) separates the contributions of sources about a target into unique, redundant, and synergistic components of information. In essence, PID answers the question of “who knows what” of a system of random variables and hence has applications to a wide spectrum of fields ranging from social to biological sciences. The paper presents MaxEnt3D_Pid, an algorithm that computes the PID of three sources, based on a recently-proposed maximum entropy measure, using convex optimization (cone programming). We describe the algorithm and its associated software utilization and report the results of various experiments assessing its accuracy. Moreover, the paper shows that a hierarchy of bivariate and trivariate PID allows obtaining the finer quantities of the trivariate partial information measure.

## 1. Introduction: Motivation and Significance

The characterization of dependencies within complex multivariate systems helps to identify the mechanisms operating in the system and understanding their function. Recent work has developed methods to characterize multivariate interactions by separating *n*-variate dependencies for different orders *n* [1,2,3,4,5]. In particular, the work of Williams and Beer [6,7] introduced a framework, called partial information decomposition (PID), which quantifies whether different input variables provide redundant, unique, or synergistic information about an output variable when combined with other input variables. Intuitively, inputs are redundant if each carries individually information about the same aspects of the output. Information is unique if it is not carried by any other single (or group of) variables, and synergistic information can only be retrieved combining several inputs.

This information-theoretic approach to study interactions has found many applications to complex systems such as gene networks (e.g., [8,9,10]), interactive agents (e.g., [11,12,13,14]), or neural processing (e.g., [15,16,17]). More generally, the nature of the information contained in the inputs determines the complexity of extracting it [18,19], how robust it is to disrupt the system [20], or how input dimensionality can be reduced without information loss [21,22].

Despite this great potential, the applicability of the PID framework has been hindered by the lack of agreement on the definition of a suitable measure of redundancy. In particular, Harder et al. [23] indicated that the original measure proposed by [6] only quantifies common amounts of information, instead of shared information that is qualitatively the same. A constellation of measures has been proposed to implement the PID (e.g., [23,24,25,26,27,28,29]), and core properties, such as requiring nonnegativity as a property of the measures, are still the subject of debate [29,30,31,32].

A widespread application of the PID framework has also been limited by the lack of multivariate implementations. Some of the proposed measures were only defined for the bivariate case [23,24,33]. Other multivariate measures allow negative components in the PID [26,29], which, although it may be adequate for statistical characterization of dependencies, limits the interpretation of the information-theoretic quantities in terms of information communication [34]. Even though at the level of local information, negativity is regarded as misinformation and can be interpreted, for example, operationally in terms of changes in belief [35], when considering information in the context of communication [36], then interpreting it as the number of messages to be retrieved without error through a noisy channel requires nonnegativity; for example, assessing the information representation about multidimensional sensory stimulus across neurons, in particular the analyses of the information content of neural responses [17,37]. Among the PID measures proposed, the maximum entropy measures of Bertschinger et al. [24] have a preeminent role in the bivariate case because they provide bounds for any other measure consistent with a set of properties shared by many of the proposed measures. Motivated by this special role of the maximum entropy measures, Chicharro [38] extended the maximum entropy approach to measures of the multivariate redundant information, which provide analogous bounds for the multivariate case. However, the work in [38] did not address their numerical implementation.

In this work, we present MaxEnt3D_Pid, a Python module that computes a trivariate information decomposition following the maximum entropy PID of [38] and exploits the connection with the bivariate decompositions associated with the trivariate ones [28]. This is, to our knowledge, the first available implementation of the maximum-entropy PID framework beyond the bivariate case [39,40,41,42], see Appendix B. This implementation is relevant for the theoretical development and practical use of the PID framework.

From a theoretical point of view, this implementation will provide the possibility to test the properties of the PID beyond the bivariate case. This is critical with regard to the nonnegativity property because, while nonnegativity is guaranteed in the bivariate case, for the multivariate case, it has been proven that negative terms can appear in the presence of deterministic dependencies [30,32,43]. However, the violation of nonnegativity has only been proven with isolated counterexamples, and it is not understood which properties of a system’s dependencies lead to negative PID measures.

From a practical point of view, the trivariate PID allows studying new types of distributed information that only appear beyond the bivariate case, such as information that is redundant for two inputs and unique with respect to a third [6]. This extension is significant both to study multivariate systems directly, as well as to be exploited for data analysis [21,44]. As mentioned above, the characterization of synergy and redundancy in multivariate systems is relevant for a broad range of fields that encompass social and biological systems. So far, the PID has particularly found applications in neuroscience (e.g., [17,37,45,46,47,48]). For data analysis, the quantification of multivariate redundancy can be applied to dimensionality reduction [22] or to better understand how representations emerge in neural networks during learning [49,50]. Altogether, this software promises to contribute significantly to the refinement of the information-theoretic tools it implements and also to foster its widespread application to analyze data from multivariate systems.

## 2. Models and Software

The section starts by briefly describing the mathematical model of the problem. Then, it discusses the architecture of MaxEnt3D_Pid. It closes by explaining in details of how to use the software.

### 2.1. Maximum Entropy Decomposition Measure

Consider X,Y, and Z as the sources and T as the target of some system. Let *P* be the joint distribution of (T,X,Y,Z) and MI(T;S) be the mutual information of T and S, where S is any nonempty subset of (X,Y,Z). The PID decomposes MI(T;X,Y,Z) into finer parts, namely synergistic, unique, redundant unique, and redundant information. These finer parts respect certain identities [6], e.g., a subset of them sums up to MI(T,X) (all identities are explained in Appendix A and Appendix C). Following the maximum entropy approach [24], to obtain this decomposition, it is necessary to solve the following optimization problems:

(1a) $$\begin{array}{cc} \min_{\Delta_{P}}\mathrm{MI}\left(T;X,Y,Z\right) & \end{array}$$

(1b) $$\begin{array}{cc} \min_{\Delta_{P}}\mathrm{MI}\left(T;X_{1},X_{2}\right) & \;\mathrm{for}\;X_{1},X_{2}\in \left\{X,Y,Z\right\} \end{array}$$

where:

$$\begin{array}{c} \Delta_{P}=\{Q\in \Delta :Q(T,X)=P(T,X),Q(T,Y)=P(T,Y),\\ Q(T,Z)=P(T,Z)\} \end{array}$$

and Δ is the set of all joint distributions of (T,X,Y,Z). The four minimization problems in Equation (1a,b) can be formulated as exponential cone programs, a special case of convex optimization. The authors refer to [41] for a nutshell introduction to cone programs, in particular the exponential ones. The full details on how to formulate (1a,b) as exponential cone programs and their convergence properties are explained in [51] (Chapter 5).

MaxEnt3D_Pid on its own returns the synergistic information and unique information collectively. In addition, with the help of the bivariate solver [39] (used in a specific way), the finer synergistic and unique information can also be extracted. Hence, the presented model obtains all the trivariate PID quantities. The full details for recovering the finer parts can be found in Appendices Appendix C and Appendix D.

### 2.2. Software Architecture and Functionality

MaxEnt3D_Pid is implemented using the standard Python syntax. The module uses an optimization software ECOS [52] to solve several optimization problems needed to compute the trivariate PID. To install the module, the ECOS Python package has to be installed, and then from the GitHub repository, the files MAXENT3D_PID.py, TRIVARIATE_SYN.py, TRIVARIATE_UNQ.py, and TRIVARIATE_QP.py must be downloaded [53].

**Figure 1:**
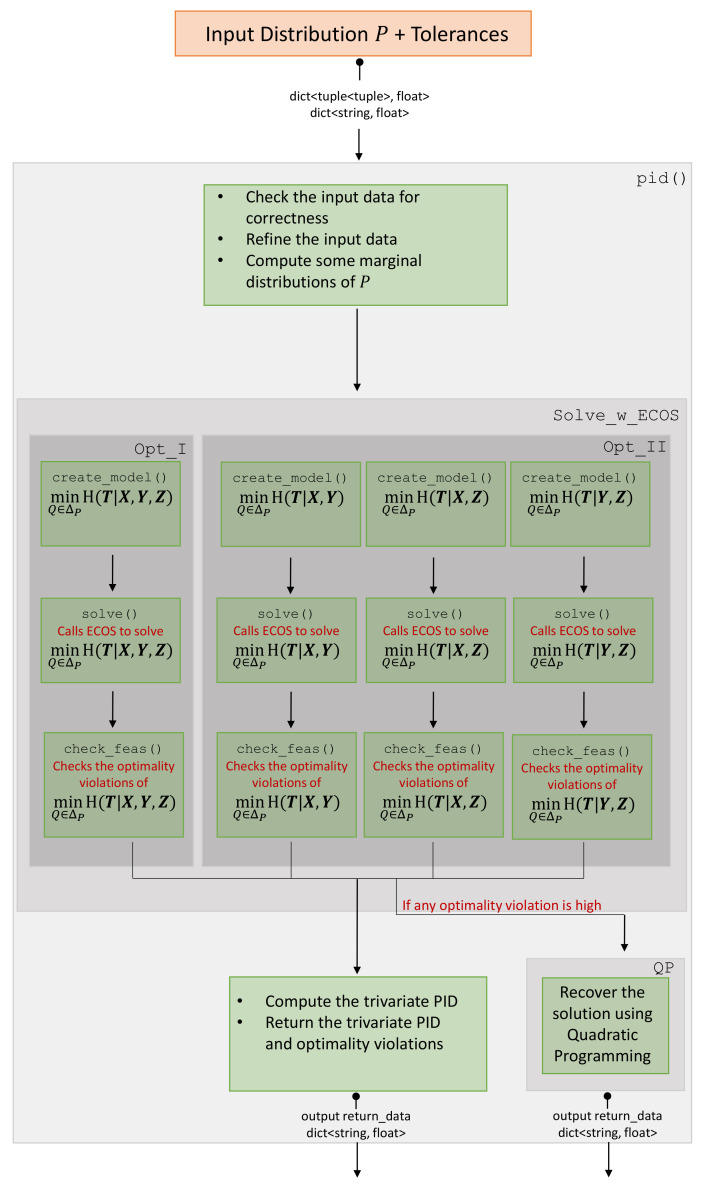
A flowchart describing the process of computing the trivariate PID via MaxEnt3D_Pid. It gives an overview of how pid() utilizes the classes Solve_w_ECOS and QP in the aim of computing the trivariate PID.

MaxEnt3D_Pid has two Python classes Solve_w_ECOS and QP. Class Solve_w_ECOS receives the marginal distributions of (T,X), (T,Y), and (T,Z) as Python dictionaries. These distributions are used by Solve_w_ECOS sub-classes Opt_I and Opt_II to solve the optimization problems of Equation (1a,b) respectively. The class QP is used to recover the solution of any optimization problems of Equation (1a,b) when Solve_w_ECOS fails to obtain a solution of a good quality. Figure 1 gives an overview of how these two classes interact.

#### 2.2.1. The Subclass Opt_I and Opt_II

The sub-classes Opt_I and Opt_II formulate the problems Equation (1a,b), use ECOS to get the optimal values, and compute their violations of the optimality certificates. They return the optimal values and their optimality violations. These violations are quality measures of the obtained PID. Figure 1 describes this process within the class Solve_w_ECOS. Note that both sub-classes Opt_I and Opt_II optimize conditional entropy functionals; however, the different number of arguments leads to a difference in how to fit the problems into the cone program and retrieving the optimal solution; hence the requirement of splitting them into different classes.

#### 2.2.2. The Class QP

Class QP acts if Solve_w_ECOS returns values of a subset of Equation (1a,b) with high optimality violations. It improves the errant values by best fitting them using quadratic programming, where the PID identities (A12) are respected.

### 2.3. Using MaxEnt3D_Pid

**Figure 2:**
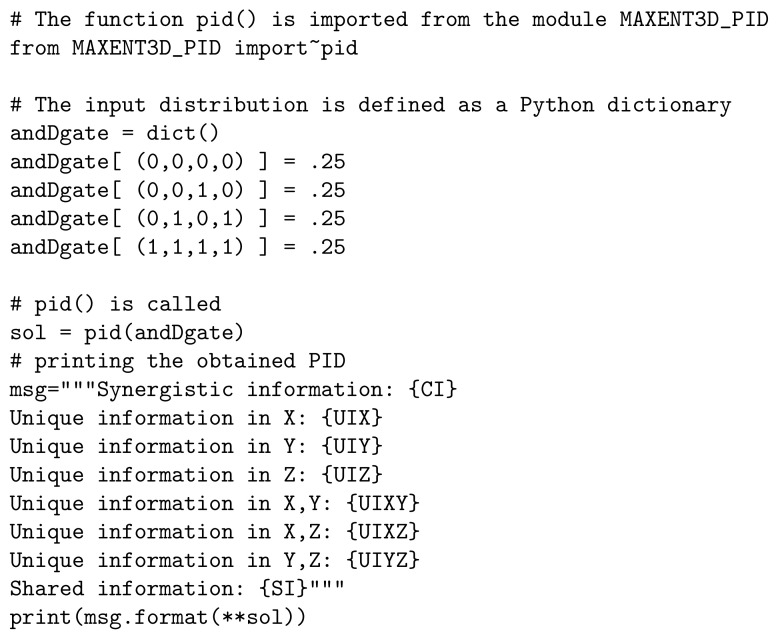
Using MaxEnt3D_Pid to compute the PID of the distribution obtained from the AndDuplicate gate (andDgate). The AndDuplicate gate evaluates T as the logical and of X and Y (X∧Y) such that Z copies X.

The process of computing the PID is packed in the function pid(). This function takes as input the distribution *P* of (T,X,Y,Z) via a Python dictionary where the tuples (t,x,y,z) are keys and their associated probability P(t,x,y,z) is the value of the key; see Figure 2. The function formulates and solves the problems of (1a,b) using Solve_w_ECOS and, if needed, uses QP to improve the solution. This function pid() returns a Python dictionary, explained in Table 1 and Table 2, containing the PID of (T,X,Y,Z) in addition to the optimality violations.

The function pid() has three other optional inputs. The first optional input is called parallel (the default value is parallel=’off’), which determines whether the process will be parallelized. If parallel=’off’, then the process is going to be done sequentially, i.e., the four problems of Equation (1a,b) are going to be formulated and solved one after the other. Their optimality violations are also computed consecutively, and then, final results are obtained; whereas, when parallel=’on’, the formulation of the four problems Equation (1a,b) is done in parallel. The four problems are solved simultaneously, and finally, the optimality violations along with the final results are computed in parallel. Thus, when parallel=’on’, there will be three sequential steps: formulating the problems, solving them, and obtaining the final results, as opposed to parallel=’off’, which requires at least twelve sequential steps.

The second optional input is a dictionary that allows the user to tune the tolerances controlling the optimization routines of ECOS listed in Table 3.

**Figure 3:**
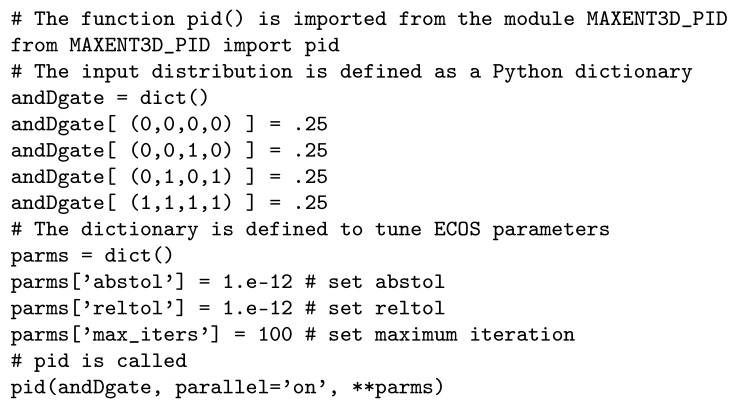
Tuning the parameters of ECOS.

In this dictionary, the user only sets the parameters that will be tuned. For example, if the user wants to achieve high accuracy, then the parameters abstol and reltol should be small (e.g., $10^{-12}$) and the parameter max_iter should be high (e.g., 1000). In Figure 3, it is shown how to modify the parameters. In this case, the solver will take longer to return the solution. For further details about the parameter’s tuning, check [41].

The third optional input is called output, and it controls what pid() will print on the user’s screen. This optional input is explained in Table 4.

**Table 4:** Description of the printing modes in the function pid().

Value	Description
0 (default)	**Simple Mode:**pid() prints its output (Python dictionary).
1	**Time Mode:** In addition to what is printed when output=0, pid() prints a flag when it starts
	preparing the optimization problems in Equation (1a,b), the total time to create each problem, a flag when it
	calls ECOS, brief stats from ECOS of each problem after solving it (Figure 4), the total time for
	retrieving the results, the total time for computing the optimality violations, and the total time
	to store the results.
2	**Detailed Time Mode:** In addition to what is printed when output=0, pid() prints for each
	problem the time of each major step of creating the model, brief stats from ECOS of each problem
	after solving it, the total time of each function used for retrieving the results, the time of each
	major step used to compute the optimality violations, the time of each function used to obtain
	the final results, and the total time to store the results.
3	**Detailed Optimization Mode:** In addition to what is printed when output=1, pid() prints
	ECOS detailed stats of each problem after solving it (Figure 5).

## 3. Illustrations

This section shows some performance tests of MaxEnt3D_Pid on three types of instances. We will describe each type of instance and show the results of testing MaxEnt3D_Pid for each one of them. The first two types, paradigmatic and Copy gates, are used as validation and memory tests. The last type, random probability distributions, is used to evaluate the accuracy and efficiency of MaxEnt3D_Pid in computing the trivariate partial information decomposition. More precisely, accuracy is evaluated as how close the values of UI(T;X∖Y,Z) and UI(T;Y∖X,Z) are to zero when Z has a considerably higher dimension, which is expected theoretically. The efficiency will be depicted in how fast MaxEnt3D_Pid is able to produce the results. The machine used comes with an Intel(R) Core(TM) i7-4790K CPU (four cores) and 16 GB of RAM. Only the computations of the last type were done using parallelization.

### 3.1. Paradigmatic Gates

As a first test, we used some trivariate PIDs that are known and have been studied previously [25]. These examples are the logic gates collected in Table 5. For these examples, the decomposition can be derived analytically, and thus, they serve to check the numerical estimations.

#### Testing

The test was implemented in test_gates.py. MaxEnt3D_Pid returns, for all gates, the same values as ([25], Table 1) up to a precision error of order $10^{-9}$. The slowest solving time (not in parallel) was one millisecond.

### 3.2. Copy Gate

As a second test, we used the Copy gate example to examine the simulation of large systems. We simulated how the solver handled large systems in terms of speed and reliability. Reliability, in this context, is meant as the consistency of the measure on large systems and the degree to which the results can be trusted to be accurate enough.

The Copy gate is the mapping of (x,y,z), chosen uniformly at random, to (t,x,y,z), where t=(x,y,z). The size of the joint distribution of (T,X,Y,Z) scales as ${\left|X\right|}^{2}\cdot {\left|Y\right|}^{2}\cdot {\left|Z\right|}^{2}$, where x,y,z∈X×Y×Z. In our test, |X|=ℓ,|Y|=m and |Z|=n, where ℓ,m,n∈{10,20,⋯,50}.

Since X,Y and Z are independent, it is easy to see that the only nonzero quantities are $\mathrm{UI}\left(T;X_{1}\setminus X_{2},X_{3}\right)=H\left(X_{1}\right)$ for $X_{1},X_{2},X_{3}\in \left\{X,Y,Z\right\}$.

#### Testing

The test was implemented in test_copy_gate.py. The slowest solving time was less than 100 s, and the worst deviation from the actual values was 0.0001%. For more details, see Table 6.

### 3.3. Random Probability Distributions

As a last example, we used joint distributions of (T,X,Y,Z) sampled uniformly at random over the probability space, to test the accuracy of the solver. The size of *T*, *X*, and *Y* was fixed to two, whereas |Z| varied in {2,⋯,14}. For each |Z|, 500 joint distributions of (T,X,Y,Z) were sampled.

#### Testing

**Figure 6:**
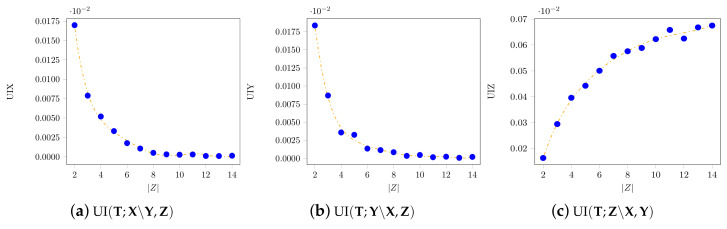
The variation of the unique information, as the size of *Z* increases, for the random probability distributions described in Section 3.3. It shows that the value of the unique information of Z increases as the dimension of *Z* increases.

**Figure 7:**
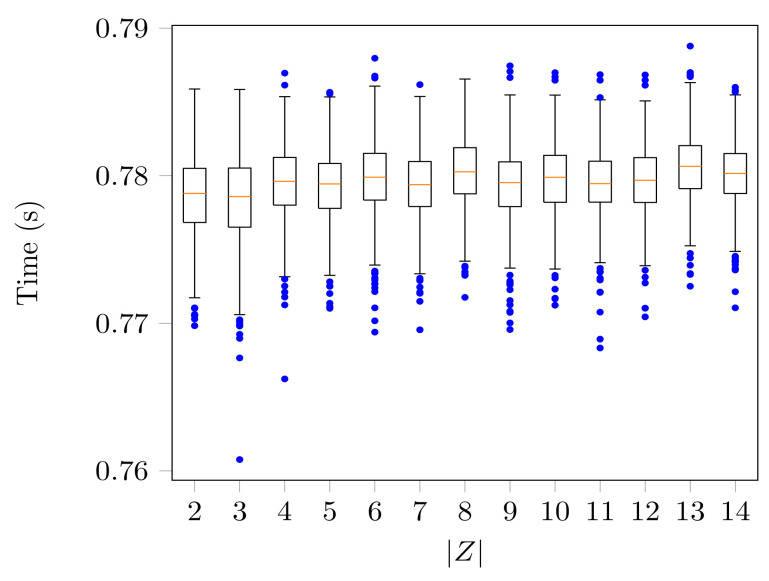
Box plotting of the time for MaxEnt3D_Pid to compute the PID of random joint probability distributions of (T,X,Y,Z) for |T|=|X|=|Y|=2 and different sizes of *Z*. For the size of the sets explored, the computational time shows a flat trend, and its variance is small.

As |Z| increased, the average value of UI(T;X∖Y,Z) and of UI(T;Y∖X,Z) decreased, while that of UI(T;Z∖X,Y) increased. In Figure 6, the accuracy of the optimization is reflected in the low divergence from zero obtained for the unique information UI(T;X∖Y,Z) and UI(T;Y∖X,Z). In Figure 7, the time has a constant trend, and the highest time value recorded was 0.8 s.

### 3.4. Challenging Distributions

We tested MaxEnt3D_Pid on randomly uniformly-sampled distributions, but with large sizes of *T*, *X*, *Y*, and *Z*. For each m, 500 joint distributions of (T,X,Y,Z) were sampled where |T|=|X|=|Y|=|Z|=m and 2≤m≤19. The idea was to check with random and huge distributions (not structured as in the case of the Copy gate) how stable the estimator was.

#### 3.4.1. Testing

For m≥5, some of the optimization problems (1a,b) did not converge due to numerical instabilities. This issue started to be frequent and significant when m≥14, for example 5% of the distributions had numerical problems in some of their optimization problems. We noticed that the returned solution from the non-convergent problem was feasible and far from optimal by a factor of 100 at most. The feasibility of the returned solution suggested fitting it along with the returned (optimal) solutions from the other convergent problems into the system of PID identities (A12), which will reduce the optimality gap.

#### 3.4.2. Recommendation

These challenging distributions have mainly two features, namely the high dimensionality of the quadruple (T,X,Y,Z) and a significant number of relatively small (almost null) probability masses along with few concentrated probability masses. We suspect that these two features combined were the main reason for the convergence problems. Our approach was to use a quadratic programming (Class QP), which focuses on reducing the optimality gap and thus returns a close PID to the optimal PID (in case of no convergence problems).

Furthermore, we advise users to mitigate such distributions by dropping some of the points with almost null probability masses. Since the objective functions in (1a,b) are continuous and smooth (full support distributions) on $\Delta_{P}$, then the PID of the mitigated distribution is considered a good approximation of that of the original distribution. Although we did not test this ad hoc on MaxEnt3D_PID, the same technique was applied to such instances for Broja_2PID ([51], Chapter 5).

We speculated that when m≥50, the solver will suffer dire numerical instabilities. It is recommended for the user to avoid large discrete binning resulting in humongous distributions.

#### 3.4.3. Time Complexity

Theoretically, Makkeh et al. [39,51] showed that the worst running time complexity for solving (1a) (the hardest problem computationally) was $O(N^{3\;/\;2}\log N)$ where N=|T×X×Y×Z|. Note that this time complexity bound was for the so-called barrier method, whereas Ecos uses the primal-dual Mehrotra predictor-corrector method [54], which does not have a theoretical complexity bound [55].

## 4. Summary and Discussion

In this work, we presented MaxEnt3D_Pid, a Python module that computes a trivariate decomposition based on the partial information decomposition (PID) framework of Williams and Beer [6], in particular following the maximum entropy PID of [38] and exploiting the connection with the bivariate decompositions associated with the trivariate one [28]. This is, to our knowledge, the first available implementation extending the maximum-entropy PID framework beyond the bivariate case [39,40,41,42].

The PID framework allows decomposing the information that a group of input variables has about a target variable into redundant, unique, and synergistic components. For the bivariate case, this results in decomposition with four components, quantifying the redundancy, synergy, and unique information of each of the two inputs. In the multivariate case, finer parts appear, which do not correspond to purely redundant or unique components. For example, the redundancy components of the multivariate decomposition can be interpreted based on local unfoldings when a new input is added, with each redundancy component unfolding into a component also redundant with the new variable and a component of unique redundancy with respect to it [38]. The PID analysis can qualitatively characterize the distribution of information beyond the standard mutual information measures [56] and has already been proven useful to study information in multivariate systems (e.g., [14,17,37,56,57,58,59,60,61,62]).

However, the definition of suited measures to quantify synergy and redundancy is still a subject of debate. From all the proposed PID measures, the maximum entropy measures by Bertschinger et al. [24] have a preeminent role in the bivariate case because they provide bounds to any other alternative measures that share fundamental properties related to the notions of redundancy and unique information. Chicharro [38] generalized the maximum entropy approach, proposing multivariate definitions of redundant information and showing that these measures implement the local unfolding of redundancy via hierarchically-related maximum entropy constraints. The package MaxEnt3D_Pid efficiently implemented the constrained information minimization operations involved in the calculation of the trivariate maximum-entropy PID decomposition. In Section 2, we described the architecture of the software, presented in detail the main function of the software that computes the PID along with its optional inputs, and described how to use it. In Section 3, we provided examples that verified that the software produced correct results on paradigmatic gates, simulated how the software scaled with large systems, and hinted to the accuracy of the software in estimating PID. In this section, we also presented challenging examples where the MaxEnt3D_PID core optimizer had convergence problems and discussed our technique to retrieve an approximate PID and some suggestions to avoid such anomalies.

The possibility to calculate a trivariate decomposition of the mutual information represents a qualitative extension of the PID framework that goes beyond an incremental extension of the bivariate case, both regarding its theoretical development and its applicability. From a theoretical point of view, regarding the maximum-entropy approach, the multivariate case requires the introduction of new types of constraints in the information minimization that do not appear in the bivariate case (Section 2 and [38]). More generally, the trivariate decomposition allows further studying one of the key unsolved issues in the PID formulation, namely the requirement of the nonnegativity of the PID measures in the multivariate case.

In particular, Harder et al. [23] indicated that the original measure proposed by [6] only quantified common amounts of information and required new properties for the PID measures, to quantify qualitatively and not quantitatively how information is distributed. However, for the multivariate case, these properties have been proven to be incompatible with guaranteeing nonnegativity, by using some counterexamples [30,32,43]. This led some subsequent proposals to define PID measures that either focus on the bivariate case [23,24] or do not require nonnegativity [26,29]. A multivariate formulation was desirable because the notions of synergy and redundancy are not restrained to the bivariate case, while nonnegativity is required for an interpretation of the measures in terms of information communication [34] and not only as a statistical description of the probability distributions. MaxEnt3D_Pid will allow systematically exploring when negative terms appear, beyond the currently-studied isolated counterexamples. Furthermore, it has been shown that in those counterexamples, the negative terms result from the criterion used to assign the information identity to different pieces of information when deterministic relations exist [32]. Therefore, a systematic analysis of the appearance of negative terms will provide a better understanding of how information identity is assigned when quantifying redundancy, which is fundamental to assess how the PID measures conform to the corresponding underlying concepts.

From a practical point of view, the trivariate decomposition allows studying qualitatively new types of distributed information, identifying finer parts of the information that the inputs have about the target, such as information that is redundant for two inputs and unique with respect to a third [6]. This is particularly useful when examining multivariate representations, such as the interactions between several genes [8,63] or characterizing the nature of coding in neural populations [64,65]. Furthermore, exploiting the connection between the bivariates and the trivariate decomposition due to the invariance of redundancy to context [28], MaxEnt3D_Pid also allows estimating the finer parts of the synergy component (Appendix D). This also offers a substantial extension in the applicability of the PID framework, in particular for the study of dynamical systems [66,67]. In particular, a question that requires a trivariate decomposition is how information transfer is distributed among multivariate dynamic processes. Information transfer is commonly quantified with the measure called transfer entropy [68,69,70,71,72], which calculates the conditional mutual information between the current state of a certain process *Y* and the past of another process *X*, given the past of *Y* and of any other processes *Z* that may also influence those two. In this case, by construction, the PID analysis should operate with three inputs corresponding to the pasts of *X*, *Y*, and *Z*. Transfer entropy is widely applied to study information flows between brain areas to characterize dynamic functional connectivity [73,74,75], and characterizing the synergy, redundancy, and unique information of these flows can provide further information about the degree of integration or segregation across brain areas [76].

More generally, the availability of software implementing the maximum entropy PID framework beyond the bivariate case promises to be useful in a wide range of fields in which interactions in multivariate systems are relevant, spanning the domain of social [12,77] and biological sciences [3,10,17,63]. Furthermore, the PID measures can also be used as a tool for data analysis and to characterize computational models. This comprises dimensionality reduction via synergy or redundancy minimization [19,22], the study of generative networks that emerge from information maximization constraints [78,79], or explaining the representations in deep networks [50].

The MaxEnt3D_Pid package presents several differences and advantages with respect to other software packages currently available to implement the PID framework. Regarding the maximum entropy approach, other packages only compute bivariate decompositions [39,40,41,42]. The dit package [42] also implements several other PID measures, including bivariate implementations for the measure of [23,27]. Among the multivariate decompositions, the ones using the measures $I_{min}$ [6] or $I_{MMI}$ [80] can readily be calculated with standard estimators of the mutual information. However, the former, as discussed above, only quantifies common amounts of information, while the latter is only valid for a certain type of data, namely multivariate Gaussian distributed. Software to estimate multivariate pointwise PIDs is also available [26,29,81]. However, as mentioned above, these measures by construction allow negative components, which may not be desirable for the interpretation of the decomposition, for example in the context of communication theory, and limits their applicability for data analysis in such regimes [22]. Altogether, MaxEnt3D_Pid is the first software that implements the mutual information PID framework via hierarchically-related maximum entropy constraints, extending the bivariate case by efficiently computing the trivariate PID measures.

## Figures and Tables

**Figure 4:**
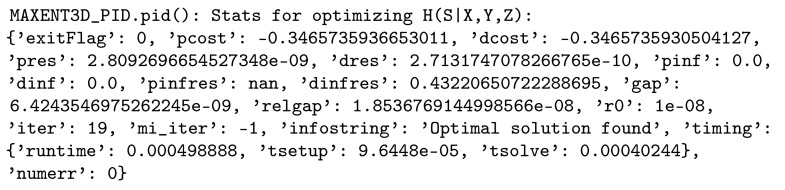
Brief stats from ECOS after solving Problem (1a).

**Figure 5:**
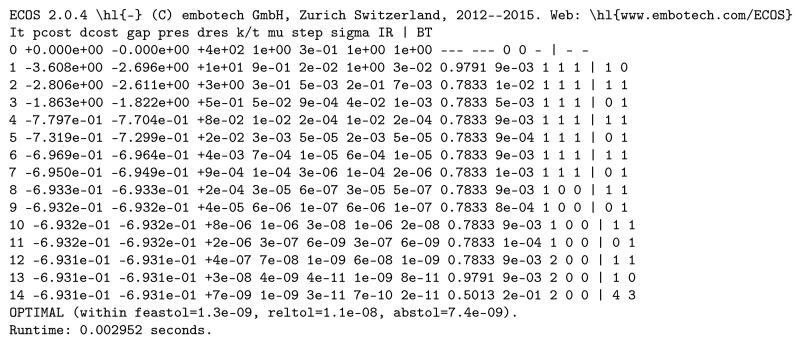
Detailed stats from ECOS after solving Problem (1a).

**Table 1:** The keys of the trivariate PID quantities in the returned dictionary. Note that $\mathrm{UI}(T;X_{i}\setminus X_{j},X_{k})$ and $\mathrm{UI}(T;X_{i},X_{k}\setminus X_{j})$ refer to unique and unique redundant information for $X_{i},X_{k},X_{j}\in \left\{X,Y,Z\right\}$, CI(T;X,Y,Z) refers to synergistic information, and SI(T;X,Y,Z) refers to redundant or shared information.

Keys	Values	Keys	Values
’UIX’	UI(T;X∖Y,Z)	’UIYZ’	UI(T;Y,Z∖X)
’UIY’	UI(T;Y∖X,Z)	’UIXZ’	UI(T;X,Z∖Y)
’UIZ’	UI(T;Z∖X,Y)	’UIXY’	UI(T;X,Y∖Z)
’CI’	CI(T;X,Y,Z)	’SI’	SI(T;X,Y,Z)

**Table 2:** The keys of optimality violations for each problem (Section 2.1a,b) in the returned dictionary.

Key	Value
’Num_Err_I’	Optimality violations of $\min_{\Delta_{P}}\mathrm{MI}\left(T;X,Y,Z\right)$
’Num_Err_12’	Optimality violations of $\min_{\Delta_{P}}\mathrm{MI}\left(T;X,Y\right)$
’Num_Err_13’	Optimality violations of $\min_{\Delta_{P}}\mathrm{MI}\left(T;X,Z\right)$
’Num_Err_23’	Optimality violations of $\min_{\Delta_{P}}\mathrm{MI}\left(T;Y,Z\right)$

**Table 3:** Parameters (tolerances) that govern the optimization in ECOS.

Parameter	Description	Default Value
feastol	primal/dual feasibility tolerance	$10^{-7}$
abstol	absolute tolerance on the duality gap	$10^{-6}$
reltol	relative tolerance on the duality gap	$10^{-6}$
feastol_inacc	primal/dual infeasibility *relaxed* tolerance	$10^{-3}$
abstol_inacc	absolute *relaxed* tolerance on the duality gap	$10^{-4}$
reltol_inacc	*relaxed* relative duality gap	$10^{-4}$
max_iter	maximum number of iterations that ECOS does	100

**Table 5:** Paradigmatic gates with a brief explanation of their operation, where ⊕ is the logical Xor and ∧ is the logical And.

Instance	Operation
XorDuplicate	T=X⊕Y;Z=X;X,Y i.i.d.
XorLoses	T=X⊕Y;Z=X⊕Y;X,Y i.i.d.
XorMultiCoal	T=U⊕V⊕W;X=(U,V),
	Y=(U,W),Z=(V,W);U,V,W i.i.d.
AndDuplicate	T=X∧Y;Z=X;X,Y i.i.d.

**Table 6:** Copy gate results. The results are divided into three sets ordered increasingly w.r.t. the size of the joint distributions. Dimensions capture the unordered triplet (|X|,|Y|,|Z|), and the deviation is computed as the maximum over all PID quantities of $100|\tilde{r}-r|$ where $\tilde{r}$ is the obtained PID quantity and *r* is the analytical PID quantity. Note that the theoretical results are either zero or $\log_{2}\left(|S|\right)$, where S∈X,Y,Z.

Set 1			Set 2			Set 3		
Dimensions	Time (s)	Deviation (%)	Dimensions	Time (s)	Deviation (%)	Dimensions	Time (s)	Deviation (%)
(10,10,10)	0.82	$10^{-7}$	(20,20,30)	7.53	$10^{-6}$	(30,30,40)	25.72	$10^{-6}$
(10,10,20)	1.06	$10^{-7}$	(10,30,50)	8.67	$10^{-6}$	(20,40,50)	24.32	$10^{-5}$
(10,10,30)	1.62	$10^{-7}$	(10,40,40)	8.68	$10^{-7}$	(30,30,50)	27.90	$10^{-6}$
(10,10,40)	2.08	$10^{-7}$	(20,20,40)	8.85	$10^{-7}$	(30,40,40)	29.85	$10^{-6}$
(10,20,20)	2.21	$10^{-7}$	(20,30,30)	11.41	$10^{-6}$	(20,50,50)	34.94	$10^{-6}$
(10,10,50)	2.61	$10^{-6}$	(10,40,50)	11.44	$10^{-6}$	(30,40,50)	47.40	$10^{-5}$
(10,20,30)	2.99	$10^{-6}$	(20,20,50)	11.34	$10^{-6}$	(40,40,40)	42.21	$10^{-5}$
(10,20,40)	4.11	$10^{-6}$	(20,30,40)	13.00	$10^{-6}$	(30,50,50)	55.60	$10^{-4}$
(20,20,20)	4.96	$10^{-6}$	(10,50,50)	16.37	$10^{-7}$	(40,40,50)	55.18	$10^{-5}$
(10,30,30)	4.43	$10^{-7}$	(30,30,30)	16.28	$10^{-7}$	(40,50,50)	89.58	$10^{-6}$
(10,20,50)	5.51	$10^{-7}$	(20,30,50)	17.24	$10^{-7}$	(50,50,50)	97.74	$10^{-5}$
(10,30,40)	6.51	$10^{-6}$	(20,40,40)	18.34	$10^{-5}$	

**Figure A1:**
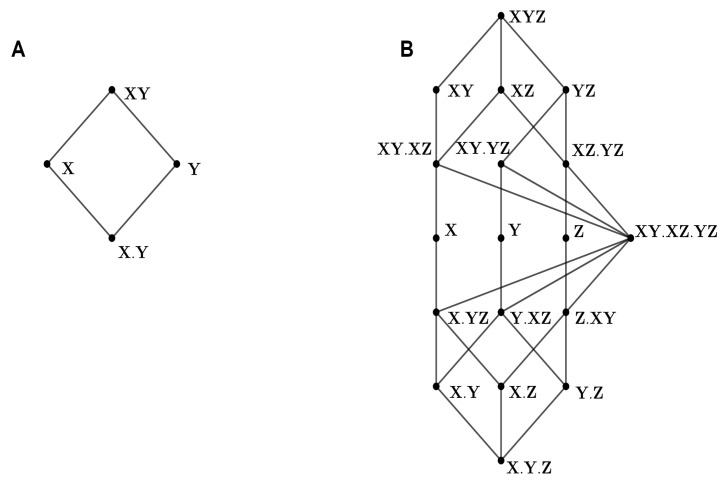
(**A**) Bivariate and (**B**) trivariate redundancy lattices. Letters indicate the mapping of terms between the lattices.

## Appendix A. Williams–Beer PID Framework

In order to decompose MI(T,S) where T is the target and S are the sources, Williams and Beer [6] defined a set of axioms leading to what is known as the redundancy lattice (Figure A1). These axioms and lattice form the framework for partial information decomposition (PID) upon which all the exiting definitions of PID are formulated.

### Appendix A.1. Williams–Beer Axioms

Suppose that a *source* *A* is a subset of S and a *collection* α is a set of sources. A shorthand notation inspired by [38] will be used to represent the collection of sources; for example, if the system is (T,X,Y,Z), then the collection of sources {{X,Y},{X,Z}} will be denoted as XY.XZ. [6] defined the following axioms that redundancy should comply with:

- Symmetry **(S)**: MI(T;α) is invariant with respect to the order of the sources in the collection.
- Self-redundancy **(SR)**: The redundancy of a collection formed by a single source is equal to the mutual information of that source.
- Monotonicity **(M)**: Adding sources to a collection can only decrease the redundancy of the resulting collection, and redundancy is kept constant when adding a superset of any of the existing sources.

### Appendix A.2. The Redundancy Lattice

Williams and Beer [6] defined a lattice formed from the collections of sources. They used **(M)** to define the partial ordering between the collections. The axiom **(S)** reflects the fact that each atom of the lattice will represent a partial information decomposition quantity. More importantly, not all the collections of sources will be considered as atoms since adding a superset of any source to the examined system does not change redundancy, i.e., **(M)**. The set of collections of sources included in the lattice which will form its atoms is defined as:

(A1) $$A\left(S\right)=\{\alpha \in P\left(S\right)-\left\{\emptyset \right\}:\forall A_{i},A_{j}\in \alpha ,A_{i}⊈A_{j}\},$$

where P(S) is the power set of S. For this set of collections (atoms), the partial ordering relation that constructs the redundancy lattice is:

(A2) ∀α,β∈A(S),(α⪯β⇔∀B∈β,∃A∈α,A⊆B),

i.e., for two collections α and β, α⪯β, if for each source in β, there is a source in α that is a subset of that source. In Figure A1, the bivariate and trivariate redundancy lattices are shown.

### Appendix A.3. Defining PID over the Redundancy Lattice

The mutual information decomposition was constructed in [6] by implicitly defining partial information measures $\delta_{C}\left(T;\alpha \right)$ associated with each node α of the redundancy lattice C (Figure A1), such that the redundancy measures are obtained as:

(A3) $$\mathrm{MI}\left(T,\alpha \right)=\sum_{\beta \in ↓\alpha }\delta_{C}\left(T;\beta \right),$$

where ↓α refers to the set of collections lower than or equal to α in the partial ordering, and hence reachable descending from α in the lattice C.

## Appendix B. Bivariate Partial Information Decomposition

Let T be the target random variable, X and Y be the two source random variables, and *P* be the joint probability distribution of (T,X,Y). The PID captures the synergistic, unique, and redundant information as follows:

- The synergistic information between X and Y about T, namely CI(T;X:Y).
- The redundant information of X and Y about T, namely SI(T;X,Y).
- The unique information of X about T, namely UI(T;X∖Y).
- The unique information of Y about T, namely UI(T;Y∖X).

This decomposition, using Beer–Williams axioms, yields these identities:

(A4) $$\begin{array}{cc} \mathrm{MI}(T;X,Y) & =\mathrm{CI}(T;X:Y)+\mathrm{SI}(T;X,Y)+\mathrm{UI}(T;X\setminus Y)+\mathrm{UI}(T;X\setminus Y)\\ \mathrm{MI}(T;X_{i}) & =\mathrm{SI}\left(T;X_{i},X_{j}\right)+\mathrm{UI}\left(T;X_{i}\setminus X_{j}\right)\;\mathrm{for}\;\mathrm{all}\;X_{i},X_{j}\in \left\{X,Y\right\}. \end{array}$$

Given the generic structure of the PID framework, the work in [24] (BROJA) defined PID measures considering the following polytope:

(A5) $$\Delta_{P}=\left\{Q\in \Delta :Q\left(T,X\right)=P\left(T,X\right),Q\left(T,Y\right)=P\left(T,Y\right)\right\},$$

where Δ is the set of all joint distributions of (T,X,Y). The work in [24] (BROJA) used the maximum entropy decomposition over $\Delta_{P}$ in order to quantify the above quantities. Moreover, BROJA assumed that the following assumptions hold.

**Assumption A1** (Lemma 3[24]). *On the bivariate redundancy lattice (Figure A1), the following assumptions must hold to quantify the PID*

1. All partial information measures of the redundancy lattice are nonnegative.
2. The terms δ(T;X.Y),δ(T;X), and δ(T;Y), are constant on $\Delta_{P}$.
3. The synergistic term, namely δ(T,XY), vanishes on $\Delta_{P}$ upon minimizing the mutual information MI(T;X,Y).

Under the above assumptions and using maximal entropy decomposition, BROJA defined the following optimization problems that compute the PID quantities.

(A6a) $$\begin{array}{cc} \tilde{\mathrm{CI}}\left(T;X:Y\right) & =\mathrm{MI}\left(T;X,Y\right)-\min_{Q\in \Delta_{P}}\mathrm{MI}\left(T;Y,X\right) \end{array}$$

(A6b) $$\begin{array}{cc} \tilde{\mathrm{UI}}\left(T;X_{i}\setminus X_{j}\right) & =\min_{Q\in \Delta_{P}}\mathrm{MI}\left(T;X_{i},X_{j}\right)-\min_{Q\in \Delta_{P}}\mathrm{MI}\left(T;X_{j}\right)\;\mathrm{for}\;\mathrm{all}\;X_{i},X_{j}\in \left\{X,Y\right\} \end{array}$$

(A6c) $$\begin{array}{cc} \tilde{\mathrm{SI}}\left(T;X,Y\right) & =\max_{Q\in \Delta_{P}}\mathrm{CoI}\left(T;X;Y\right) \end{array}$$

where CoI(T;X;Y) is the co-information of T,X, and Y defined as MI(T,X)−MI(T,X∣Y). Note that [38] proved that (A6c) is equivalent to:

$$\tilde{\mathrm{SI}}\left(T;X,Y\right)=\min_{\begin{array}{c} Q\in \Delta_{P},\\ \mathrm{CoI}(T;X;Y)=0 \end{array}}\mathrm{MI}\left(T;X,Y\right)-\min_{Q\in \Delta_{P}}\mathrm{MI}\left(T;X,Y\right).$$

### Appendix B.1. Mutual Information over the Bivariate Redundancy Lattice

This subsection writes down some mutual information quantities in terms of redundancy lattice partial information measures using (A3). These formulas will be used in the following subsection to verify that the measures defined in (A6a–c) to quantify the desired partial information quantities. MI(T;X,Y) will be the sum of partial information measure on every node of the redundancy lattice C as follows:

(A7) $$\begin{array}{cc} \mathrm{MI}(T;X,Y) & =\delta (T,XY)+\delta (T,X)+\delta (T,Y)+\delta (T,X.Y). \end{array}$$

The mutual information of one source and the target is expressed as:

(A8) $$\begin{array}{cc} \mathrm{MI}(T;X_{i}) & =\delta \left(T,X_{i}\right)+\delta \left(T,X_{i}.X_{j}\right)\;\mathrm{for}\;X_{i},X_{j}\in \left\{X,Y\right\}. \end{array}$$

The mutual information of one source and the target conditioned on knowing the other source is expressed as:

(A9) $$\begin{array}{cc} \mathrm{MI}(T;X_{i}|X_{j}) & =\delta \left(T,X_{i}X_{j}\right)+\delta \left(T,X_{i}\right)\;\mathrm{for}\;\mathrm{all}\;X_{i},X_{j}\in \left\{X,Y\right\}. \end{array}$$

The co-information CoI(T;X;Y) is expressed as:

(A10) $$\begin{array}{cc} \mathrm{CoI}(T;X;Y) & =\delta (T,X.Y)-\delta (T,XY). \end{array}$$

### Appendix B.2. Verification of BROJA Optimization

This subsection will verify that the measures defined in (A6a–c) quantify the desired partial information quantities under the maximum decomposition principle. Assumption A1 implies that $\min_{Q\in \Delta_{P}}\delta \left(T,XY\right)=0,$$\min_{Q\in \Delta_{P}}\delta \left(T,X.Y\right)=\delta \left(T,1.2\right),\min_{Q\in \Delta_{P}}\delta \left(T,X\right)=\delta \left(T,X\right)$, and $\min_{Q\in \Delta_{P}}\delta \left(T,Y\right)=\delta \left(T,Y\right)$. Therefore, it is easy to see that:

$$\begin{array}{cc} \tilde{\mathrm{CI}}\left(T;X:Y\right) & =\mathrm{MI}\left(T;X,Y\right)-\min_{Q\in \Delta_{P}}\mathrm{MI}\left(T;Y,X\right)=\delta \left(T,XY\right)\\ \tilde{\mathrm{UI}}\left(T;X\setminus Y\right) & =\min_{Q\in \Delta_{P}}\mathrm{MI}\left(T;X,Y\right)-\min_{Q\in \Delta_{P}}\mathrm{MI}\left(T;Y\right)=\delta \left(T,X\right)\\ \tilde{\mathrm{UI}}\left(T;Y\setminus X\right) & =\min_{Q\in \Delta_{P}}\mathrm{MI}\left(T;X,Y\right)-\min_{Q\in \Delta_{P}}\mathrm{MI}\left(T;X\right)=\delta \left(T,Y\right). \end{array}$$

Now, CoI(T;X;Y)=0 implies that δ(T,XY)=δ(T,X.Y); thus:

$$\begin{array}{cc} \tilde{\mathrm{SI}}\left(T;X,Y\right) & =\min_{\begin{array}{c} Q\in \Delta_{P},\\ \mathrm{CoI}(T;X;Y)=0 \end{array}}\mathrm{MI}\left(T;X,Y\right)-\min_{Q\in \Delta_{P}}\mathrm{MI}\left(T;X,Y\right)=\delta \left(T,X.Y\right). \end{array}$$

Hence, under Assumption A1,

(A11) $$\begin{array}{cc} \; & \tilde{\mathrm{CI}}\left(T;X:Y\right)=\mathrm{CI}\left(T;X:Y\right),\;\tilde{\mathrm{UI}}\left(T;X\setminus Y\right)=\mathrm{UI}\left(T;X\setminus Y\right)\\ \; & \tilde{\mathrm{UI}}\left(T;Y\setminus X\right)=\mathrm{UI}\left(T;Y\setminus X\right),\;\tilde{\mathrm{SI}}\left(T;X,Y,Z\right)=\mathrm{SI}\left(T;X,Y,Z\right). \end{array}$$

## Appendix C. Maximum Entropy Decomposition of Trivariate PID

Let T be the target random variable, X,Y,Z be the source random variables, and *P* be the joint probability distribution of (T,X,Y,Z). Chicharro [38] using maximum entropy decomposed mutual information MI(T,X,Y,Z) into: synergistic, unique, unique redundant, and redundant information. In this decomposition,

- the synergistic quantity, $\tilde{\mathrm{CI}}\left(T;X,Y,Z\right)$, captures the sum of all individual synergistic terms, namely δ(T;XYZ)+δ(T;XY)+δ(T;XZ)+δ(T;YZ)+δ(T;XY.XZ)+δ(T;XY.YZ)+δ(T;XZ.YZ)+δ(T;XY.XZ.YZ),
- the unique information, $\tilde{\mathrm{UI}}\left(T;X_{i}\setminus X_{j},X_{k}\right)$, captures the sum of the information that $X_{i}$ has about T solely, $\delta (T;X_{i})$, and the information $X_{i}$ knows redundantly with the synergy of $\left(X_{j},X_{k}\right)$, $\delta (T;X_{i}.X_{j}X_{k})$ for all $X_{i},X_{j},X_{k}\in \left\{X,Y,Z\right\}$,
- the unique redundant information, $\tilde{\mathrm{UI}}\left(T;X_{i},X_{j}\setminus X_{k}\right)$, captures the actual unique information that $X_{i}$ and $X_{j}$ have redundantly about T, $\delta (T;X_{i}.X_{j})$ for all $X_{i},X_{j},X_{k}\in \left\{X,Y,Z\right\}$,
- and the redundant information, $\tilde{\mathrm{SI}}\left(T;X,Y,Z\right)$ captures the actual redundant information of X,Y, and Z about T, i.e, δ(T;X.Y.Z).

Using Beer–Williams axioms. the decomposition yields these identities:

(A12) $$\begin{array}{cc} \mathrm{MI}(T;X,Y,Z) & =\tilde{\mathrm{CI}}\left(T;X,Y,Z\right)+\tilde{\mathrm{SI}}\left(T;X;Y;Z\right)+\tilde{\mathrm{UI}}\left(T;X\setminus Y,Z\right)+\tilde{\mathrm{UI}}\left(T;Y\setminus X,Z\right)\\ \; & +\tilde{\mathrm{UI}}\left(T;Z\setminus X,Y\right)+\tilde{\mathrm{UI}}\left(T;X,Y\setminus Z\right)+\tilde{\mathrm{UI}}\left(T;X,Z\setminus Y\right)+\tilde{\mathrm{UI}}\left(T;Y,Z\setminus X\right)\\ \mathrm{MI}(T;X_{i}) & =\tilde{\mathrm{SI}}\left(T;X_{i};X_{j};X_{k}\right)+\tilde{\mathrm{UI}}\left(T;X_{i}\setminus X_{j},X_{k}\right)+\tilde{\mathrm{UI}}\left(T;X_{i},X_{j}\setminus X_{k}\right)\\ \; & +\tilde{\mathrm{UI}}\left(T;X_{i},X_{k}\setminus X_{j}\right)\;\mathrm{for}\;\mathrm{all}\;X_{i},X_{j},X_{k}\in \left\{X,Y,Z\right\}. \end{array}$$

and Δ is the set of all joint distributions of (T,X,Y,Z). The measure uses the maximum entropy decomposition over $\Delta_{P}$ in order to compute the above quantities. Moreover, the work in [38] made some assumptions over the partial information measures of the redundancy lattice.

**Assumption A2** (Assumptions a.1 and a.2 in [38]). *On the trivariate redundancy lattice (Figure A1), the following assumptions are made to quantify the PID*

1. All partial information measures of the redundancy lattice are nonnegative.
2. The terms δ(T;X.Y.Z) and $\delta (T;X_{i}.X_{j})$ for all $X_{i},X_{j}\in \left\{X,Y,Z\right\}$ are invariant on $\Delta_{P}$.
3. The summands $\delta \left(T;X_{i}\right)+\delta \left(T;X_{i}.X_{j}X_{k}\right)$ for all $X_{i},X_{j},X_{k}\in \left\{X,Y,Z\right\}$ are invariant on $\Delta_{P}$.
4. The terms $\delta \left(T;XYZ\right),\delta \left(T;XY.XZ.YZ\right),\delta \left(T;X_{i}X_{j}\right),\delta \left(T;X_{i}X_{j}.X_{i}X_{k}\right),\delta \left(T;X_{i}\right)$, and $\delta (T;X_{i}.X_{j}X_{k})$ for all $X_{i},X_{j},X_{k}\in \left\{X,Y,Z\right\}$ are not constant on $\Delta_{P}$.
5. All synergistic terms, δ(T;XYZ),δ(T;XY.XZ.YZ),$\delta (T;X_{i}X_{j}),$ and $\delta (T;X_{i}X_{j}.X_{i}X_{k})$ for all $X_{i},X_{j},X_{k}\in \left\{X,Y,Z\right\}$ vanish at the minimum over $\Delta_{P}$.
6. The partial information measures $\delta (T;X_{i}.X_{j}X_{k})$ for all $X_{i},X_{j},X_{k}\in \left\{X,Y,Z\right\}$ vanish at the minimum over $\Delta_{P}$.

Under the above assumptions and using maximal entropy decomposition, the work in [38] defined the following optimization problems that compute the PID quantities.

(A13a) $$\begin{array}{cc} \tilde{\mathrm{CI}}\left(T;X,Y,Z\right) & =\mathrm{MI}\left(T;X,Y,Z\right)-\min_{Q\in \Delta_{P}}\mathrm{MI}\left(T;Y,X,Z\right) \end{array}$$

(A13b) $$\begin{array}{cc} \tilde{\mathrm{UI}}\left(T;X_{i}\setminus X_{j},X_{k}\right) & =\min_{Q\in \Delta_{P}}\mathrm{MI}\left(T;X_{i},X_{j},X_{k}\right)-\min_{Q\in \Delta_{P}}\mathrm{MI}\left(T;X_{j},X_{k}\right)\\ \; & \mathrm{for}\;\mathrm{all}\;X_{i},X_{j},X_{k}\in \left\{X,Y,Z\right\} \end{array}$$

(A13c) $$\begin{array}{cc} \tilde{\mathrm{UI}}\left(T;X_{i},X_{j}\setminus X_{k}\right) & =\min_{\begin{array}{c} Q\in \Delta_{P},\\ \mathrm{CoI}(T;X_{i};X_{j}|X_{k})=0 \end{array}}\mathrm{MI}\left(T;X_{i},X_{j},X_{k}\right)-\min_{Q\in \Delta_{P}}\mathrm{MI}\left(T;X_{i},X_{j},X_{k}\right)\\ \; & \mathrm{for}\;\mathrm{all}\;X_{i},X_{j},X_{k}\in \left\{X,Y,Z\right\} \end{array}$$

(A13d) $$\begin{array}{cc} \tilde{\mathrm{SI}}\left(T;Z,Y,Z\right) & =\min_{\begin{array}{c} Q\in \Delta_{P},\mathrm{CoI}\left(T;X;Y\right)=0,\\ \mathrm{CoI}(T;X;Y|Z)=0,w(Q) \end{array}}\mathrm{MI}\left(T;X,Y,Z\right)-\min_{\begin{array}{c} Q\in \Delta_{P},w\left(Q\right),\\ \mathrm{CoI}(T;X;Y|Z)=0 \end{array}}\mathrm{MI}\left(T;X,Y,Z\right), \end{array}$$

where:

$$\begin{array}{cc} w(Q):=\{Q\in \Delta : & \mathrm{MI}\left(T;X,Y\right)=\min_{Q\in \Delta_{P}}\mathrm{MI}\left(T;X,Y\right),\mathrm{MI}\left(T;X,Z\right)=\min_{Q\in \Delta_{P}}\mathrm{MI}\left(T;X,Z\right),\\ \; & \mathrm{MI}\left(T;Y,Z\right)=\min_{Q\in \Delta_{P}}\mathrm{MI}\left(T;Y,Z\right)\}. \end{array}$$

### Mutual Information over the Trivariate Redundancy Lattice

This subsection writes down some mutual information quantities in terms of the trivariate redundancy lattice’s partial information measures using (A3). The verification that the optimization defined in (A13a–d) quantifies the desired partial information quantities was discussed in detail by [38] and so will be skipped. However, these formulas are needed later when discussing how to compute the individual PID terms using a hierarchy of BROJA and [38] PID decompositions. The mutual information quantities are in terms of redundancy lattice partial information measures.

MI(T;X,Y,Z) will be the sum of the partial information measure on every node of the redundancy lattice C as follows.

(A14) $$\begin{array}{cc} \mathrm{MI}(T;X,Y,Z) & =\delta (T,XYZ)+\delta (T,XY)+\delta (T,XZ)+\delta (T,YZ)+\delta (T,XY.XZ)\\ \; & +\delta (T,XY.YZ)+\delta (T,XZ.YZ)+\delta (T,XY.XZ.YZ)+\delta (T,X)\\ \; & +\delta (T,Y)+\delta (T,Z)+\delta (T,X.YZ)+\delta (T,Y.XZ)+\delta (T,Z.XY)\\ \; & +\delta (T,X.Y)+\delta (T,X.Z)+\delta (T,Y.Z)+\delta (T,X.Y.Z). \end{array}$$

For all $X_{i},X_{j},X_{k}\in \left\{X,Y,Z\right\},$ the mutual information of two sources (jointly) and the target is expressed as:

(A15) $$\begin{array}{cc} \mathrm{MI}(T;X_{i},X_{j}) & =\delta \left(T,X_{i}X_{j}\right)+\delta \left(T,X_{i}X_{j}.X_{i}X_{k}\right)+\delta \left(T,X_{i}X_{j}.X_{j}X_{k}\right)\\ \; & +\delta \left(T,X_{i}X_{j}.X_{i}X_{k}.X_{j}X_{k}\right)+\delta \left(T,X_{i}\right)+\delta \left(T,X_{j}\right)+\delta \left(T,X_{i}.X_{j}X_{k}\right)\\ \; & +\delta \left(T,X_{j}.X_{i}X_{k}\right)+\delta \left(T,X_{k}.X_{i}X_{j}\right)+\delta \left(T,X_{i}.X_{j}\right)+\delta \left(T,X_{i}.X_{k}\right)\\ \; & +\delta \left(T,X_{j}.X_{k}\right)+\delta \left(T,X_{i}.X_{j}.X_{k}\right). \end{array}$$

For all $X_{i},X_{j},X_{k}\in \left\{X,Y,Z\right\},$ the mutual information of one source and the target is as follows:

(A16) $$\begin{array}{cc} \mathrm{MI}(T;X_{i}) & =\delta \left(T,X_{i}\right)+\delta \left(T,X_{i}.X_{j}X_{k}\right)+\delta \left(T,X_{i}.X_{j}\right)+\delta \left(T,X_{i}.X_{k}\right)+\delta \left(T,X_{i}.X_{j}.X_{k}\right). \end{array}$$

For all $X_{i},X_{j},X_{k}\in \left\{X,Y,Z\right\},$ the mutual information of two sources (jointly) and the target conditioned on knowing the other source is evaluated as:

(A17) $$\begin{array}{cc} \mathrm{MI}(T;X_{i},X_{j}|X_{k}) & =\delta \left(T,X_{i}X_{j}X_{k}\right)+\delta \left(T,X_{i}X_{j}\right)+\delta \left(T,X_{i}X_{k}\right)+\delta \left(T,X_{j}X_{k}\right)\\ \; & +\delta \left(T,X_{i}X_{j}.X_{i}X_{k}\right)+\delta \left(T,X_{i}X_{j}.X_{j}X_{k}\right)+\delta \left(T,X_{i}X_{k}.X_{j}X_{k}\right)\\ \; & +\delta \left(T,X_{i}X_{j}.X_{i}X_{k}.X_{j}X_{k}\right)+\delta \left(T,X_{i}\right)\\ \; & +\delta \left(T,X_{j}\right)+\delta \left(T,X_{i}.X_{j}X_{k}\right)+\delta \left(T,X_{j}.X_{i}X_{k}\right)+\delta \left(T,X_{i}.X_{j}\right). \end{array}$$

For all $X_{i},X_{j},X_{k}\in \left\{X,Y,Z\right\},$ the mutual information of one source and the target conditioned on knowing only one of the other sources is written as:

(A18) $$\begin{array}{cc} \mathrm{MI}(T;X_{i}|X_{j}) & =\delta \left(T,X_{i}X_{j}\right)+\delta \left(T,X_{i}X_{j}.X_{i}X_{k}\right)+\delta \left(T,X_{i}X_{j}.X_{j}X_{k}\right)\\ \; & +\delta \left(T,X_{i}X_{j}.X_{i}X_{k}.X_{j}X_{k}\right)+\delta \left(T,X_{i}\right)+\delta \left(T,X_{i}.X_{j}X_{k}\right)\\ \; & +\delta \left(T,X_{k}.X_{i}X_{j}\right)+\delta \left(T,X_{i}.X_{k}\right). \end{array}$$

For all $X_{i},X_{j},X_{k}\in \left\{X,Y,Z\right\},$ the mutual information of one source and the target conditioned on knowing the other sources is:

(A19) $$\begin{array}{cc} \mathrm{MI}(T;X_{i}|X_{j},X_{k}) & =\delta \left(T,X_{i}X_{j}X_{k}\right)+\delta \left(T,X_{i}X_{j}\right)+\delta \left(T,X_{i}X_{k}\right)\\ \; & +\delta \left(T,X_{i}X_{j}.X_{i}X_{k}\right)+\delta \left(T,X_{i}\right). \end{array}$$

For all $X_{i},X_{j},X_{k}\in \left\{X,Y,Z\right\},$ the co-information of two sources and the target is expressed as:

(A20) $$\begin{array}{cc} \mathrm{CoI}(T;X_{i};X_{j}) & =\delta \left(T,X_{i}.X_{j}\right)+\delta \left(T,X_{i}.X_{j}.X_{k}\right)-(\delta \left(T,X_{i}X_{j}\right)+\delta \left(T,X_{i}X_{j}.X_{i}X_{k}\right)\\ \; & +\delta \left(T,X_{i}X_{j}.X_{j}X_{k}\right)+\delta \left(T,X_{i}X_{j}.X_{i}X_{k}.X_{j}X_{k}\right)+\delta \left(T,X_{k}.X_{i}X_{j}\right)). \end{array}$$

For all $X_{i},X_{j},X_{k}\in \left\{X,Y,Z\right\},$ the co-information of one source, two sources (jointly), and the target is as follows:

(A21) $$\begin{array}{cc} \mathrm{CoI}(T;X_{i};X_{j},X_{k}) & =\delta \left(T,X_{i}.X_{j}X_{k}\right)+\delta \left(T,X_{i}.X_{j}\right)+\delta \left(T,X_{i}.X_{k}\right)+\delta \left(T,X_{i}.X_{j}.X_{k}\right)\\ \; & -\left(\delta \left(T,X_{i}X_{j}X_{k}\right)+\delta \left(T,X_{i}X_{j}\right)+\delta \left(T,X_{i}X_{k}\right)+\delta \left(T,X_{i}X_{j}.X_{i}X_{k}\right)\right). \end{array}$$

For all $X_{i},X_{j},X_{k}\in \left\{X,Y,Z\right\},$ the co-information of two sources (jointly), two sources (jointly), and the target is evaluated as:

(A22) $$\begin{array}{cc} \mathrm{CoI}(T;X_{i},X_{j};X_{i},X_{k}) & =\delta \left(T,X_{i}X_{j}.X_{i}X_{k}\right)+\delta \left(T,X_{i}X_{j}.X_{i}X_{k}.X_{j}X_{k}\right)+\delta \left(T,X_{i}\right)\\ \; & +\delta \left(T,X_{k}.X_{i}X_{j}\right)+\delta \left(T,X_{i}.X_{j}\right)+\delta \left(T,X_{i}.X_{j}X_{k}\right)+\delta \left(T,X_{j}.X_{i}X_{k}\right)\\ \; & +\delta \left(T,X_{i}.X_{k}\right)+\delta \left(T,X_{j}.X_{k}\right)+\delta \left(T,X_{i}.X_{j}.X_{k}\right)-\delta \left(T,X_{i}X_{j}X_{k}\right)\\ \; & -\delta (T,X_{j}X_{k}). \end{array}$$

For all $X_{i},X_{j},X_{k}\in \left\{X,Y,Z\right\},$ the co-information of two sources and the target conditioning on knowing the other source can be written as:

(A23) $$\begin{array}{cc} \mathrm{CoI}(T;X_{i};X_{j}|X_{k}) & =\delta \left(T,X_{i}X_{k}.X_{j}X_{k}\right)+\delta \left(T,X_{i}X_{j}.X_{i}X_{k}.X_{j}X_{k}\right)+\delta \left(T,X_{i}.X_{j}X_{k}\right)\\ \; & +\delta \left(T,X_{j}.X_{i}X_{k}\right)+\delta \left(T,X_{i}.X_{j}\right)-\delta \left(T,X_{i}X_{j}X_{k}\right)-\delta \left(T,X_{i}X_{k}\right). \end{array}$$

## Appendix D. The Finer Quantities of Trivariate Maximum Entropy PID

In Appendix C, the maximum entropy decomposition for trivariate PID returns a synergistic term, which is the sum of all individual synergy quantities, and a unique term, which is the sum of unique and unique redundancy quantities. This section aims to show how to use maximum entropy decomposition for bivariate PID in order to obtain each individual synergy quantity, as well as each individual unique and unique redundancy quantity.

Let T be the target random variable, X,Y,Z be the source random variables, and *P* be the joint probability distribution of (T,X,Y,Z). Now, BROJA will be applied to some subsystems of (T,X,Y,Z), namely, $(T,\left(X_{i},X_{j}\right),X_{k}),$ (one single source) and $\left(T,\left(X_{i},X_{j}\right),\left(X_{i},X_{k}\right)\right)$ (two double sources) for all $X_{i},X_{j},X_{k}\in \left\{X,Y,Z\right\}$. Not that the pairs $\left(X_{i},X_{j}\right)$ and $\left(X_{i},X_{k}\right)$ are ordered alphabetically. Consider the following probability polytopes upon which the optimization will be carried out:

(A24) $$\begin{array}{cc} \Delta_{P} & =\{Q\in \Delta ;Q(T,X)=P(T,X),Q(T,Y)=P(T,Y),Q(T,Z)=P(T,Z)\}\\ \Delta_{P}^{X_{i},X_{j}.X_{k}} & =\{Q\in \Delta ;Q\left(T,X_{i},X_{j}\right)=P\left(T,X_{i},X_{j}\right),Q\left(T,X_{k}\right)=P\left(T,X_{k}\right)\}\\ \; & \;\mathrm{where}\;X_{i}\neq X_{j},X_{i}\neq X_{k},X_{j}\neq X_{k}\;\mathrm{for}\;\mathrm{all}\;X_{i},X_{j},X_{k}\in \left\{X,Y,Z\right\}\\ \Delta_{P}^{X_{i},X_{j}.X_{i},X_{k}} & =\{Q\in \Delta ;Q\left(T,X_{i},X_{j}\right)=P\left(T,X_{i},X_{j}\right),Q\left(T,X_{i},X_{k}\right)=P\left(T,X_{i},X_{k}\right)\}\\ \; & \;\mathrm{where}X_{i}\neq X_{j},X_{i}\neq X_{k},X_{j}\neq X_{k}\;\mathrm{for}\;\mathrm{all}\;X_{i},X_{j},X_{k}\in \left\{X,Y,Z\right\}. \end{array}$$

Note that $\Delta_{P}⊊\Delta_{P}^{X_{i},X_{j}.X_{k}}⊊\Delta_{P}^{X_{i},X_{j}.X_{i},X_{k}}$ for all $X_{i},X_{j},X_{k}\in \left\{X,Y,Z\right\}$.

### Appendix D.1. One Single Source Subsystems

These subsystems have the form $\left(T,\left(X_{i},X_{j}\right),X_{k}\right)$ where $X_{i},X_{j},X_{k}\in \left\{X,Y,Z\right\}$, $X_{i}\neq X_{j}\neq X_{k},$ and $X_{i}\neq X_{k}$. Now, apply the BROJA decomposition to the subsystem (T,(X,Y),Z). Therefore, its four PID quantities are defined as follows:

$$\begin{array}{cc} \tilde{\mathrm{CI}}\left(T;X,Y\right) & =\mathrm{MI}\left(T;X,Y,Z\right)-\min_{Q\in \Delta_{P}^{XY.Z}}\mathrm{MI}\left(T;X,Y,Z\right)\\ \tilde{\mathrm{UI}}\left(T;X,Y\setminus Z\right) & =\min_{Q\in \Delta_{P}^{XY.Z}}\mathrm{MI}\left(T;X,Y,Z\right)-\min_{Q\in \Delta_{P}^{XY.Z}}\mathrm{MI}\left(T;Z\right)\\ \tilde{\mathrm{UI}}\left(T;Z\setminus X,Y\right) & =\min_{Q\in \Delta_{P}^{XY.Z}}\mathrm{MI}\left(T;X,Y,Z\right)-\min_{Q\in \Delta_{P}^{XY.Z}}\mathrm{MI}\left(T;X,Y\right)\\ \tilde{\mathrm{SI}}\left(T;X,Y,Z\right) & =\min_{\begin{array}{c} Q\in \Delta_{P}^{XY.Z},\\ \mathrm{CoI}(T;X,Y;Z)=0 \end{array}}\mathrm{MI}\left(T;X,Y,Z\right)-\min_{Q\in \Delta_{P}^{XY.Z}}\mathrm{MI}\left(T;X,Y,Z\right). \end{array}$$

Note that the (X,Y) marginal distribution is fixed. This implies that the mutual information MI(T;X,Y),MI(T;X∣Y),MI(T;Y∣X), and CoI(T;X;Y) is invariant over $\Delta_{P}^{XY.Z}$. Therefore, the summands δ(T,XY)+δ(T,XY.XZ)+δ(T,XY.YZ)+δ(T,XY.XZ.YZ)+δ(T,Z.XY) are fixed. However, from Assumption A2 and the fact that the X,Y marginal is fixed, the redundancy δ(T;Z.XY) is invariant over $\Delta_{P}^{XY.Z}$. Thus, in addition to 2 in Assumption A2, the following partial information measures are invariant over $\Delta_{P}^{XY.Z}$.

1. δ(T;Z.XY) since the (X,Y) marginal is fixed.
2. δ(T;Z) since MI(T;Z) and δ(T;Z.XY) are invariant over $\Delta_{P}^{XY.Z}$.
3. δ(T,XY)+δ(T,XY.XZ)+δ(T,XY.YZ)+δ(T,XY.XZ.YZ) since CoI(T;X,Y) and δ(T;Z.XY) are invariant over $\Delta_{P}^{XY.Z}$.

Thus, using Assumption A2 and the definition of MI(T;X,Y,Z) over the redundancy lattice,

$$\begin{array}{cc} \min_{Q\in \Delta_{P}^{XY.Z}}\mathrm{MI}\left(T;X,Y,Z\right) & =\delta (T,XY)+\delta (T,XY.XZ)+\delta (T,XY.YZ)+\delta (T,XY.XZ.YZ)\\ \; & +\delta (T,X)+\delta (T,Y)+\delta (T,Z)+\delta (T,X.YZ)+\delta (T,Y.XZ)\\ \; & +\delta (T,Z.XY)+\delta (T,X.Y)+\delta (T,X.Z)+\delta (T,Y.Z)+\delta (T,X.Y.Z). \end{array}$$

The synergy is evaluated as:

$$\begin{array}{cc} \tilde{\mathrm{CI}}\left(T;X,Y\right) & =\mathrm{MI}\left(T;X,Y,Z\right)-\min_{Q\in \Delta_{P}^{XY.Z}}\mathrm{MI}\left(T;X,Y,X\right)\\ \; & =\delta (T,XYZ)+\delta (T,XZ)+\delta (T,YZ)+\delta (T,XZ.YZ). \end{array}$$

The unique information of (X,Y) in terms of the redundancy lattice atoms is:

$$\begin{array}{cc} \tilde{\mathrm{UI}}\left(T;XY\setminus Z\right) & =\min_{Q\in \Delta_{P}^{XY.Z}}\mathrm{MI}\left(T;X,Y,Z\right)-\min_{Q\in \Delta_{P}^{XY.Z}}\mathrm{MI}\left(T;Z\right)\\ \; & =\delta (T,XY)+\delta (T,XY.XZ)+\delta (T,XY.YZ)+\delta (T,XY.XZ.YZ)\\ \; & +\delta (T,X)+\delta (T,Y)+\delta (T,X.YZ)+\delta (T,Y.XZ)+\delta (T,X.Y). \end{array}$$

The unique information of Z is written as:

$$\begin{array}{cc} \tilde{\mathrm{UI}}\left(T;Z\setminus XY\right) & =\min_{Q\in \Delta_{P}^{XY.Z}}\mathrm{MI}\left(T;X,Y,Z\right)-\min_{Q\in \Delta_{P}^{XY.Z}}\mathrm{MI}\left(T;X,Y\right)=\delta \left(T,Z\right) \end{array}$$

When CoI(T;X,Y;Z)=0, then δ(T,Z.XY)+δ(T,X.Z)+δ(T,Y.Z)+δ(T,X.Y.Z) is equal to δ(T,XYZ)+δ(T,XZ)+δ(T,YZ)+δ(T,XZ.YZ).

Then, in terms of redundancy lattice atoms, the shared information of (X,Y) and Z is:

$$\begin{array}{cc} \tilde{\mathrm{SI}}\left(T;X,Y,Z\right) & =\min_{\begin{array}{c} Q\in \Delta_{P}^{XY.Z},\\ \mathrm{CoI}(T;X,Y;Z)=0 \end{array}}\mathrm{MI}\left(T;X,Y,Z\right)-\min_{Q\in \Delta_{P}^{XY.Z}}\mathrm{MI}\left(T;X,Y,Z\right)\\ \; & =\delta (T,Z.XY)+\delta (T,X.Z)+\delta (T,Y.Z)+\delta (T,X.Y.Z). \end{array}$$

Hence, for all $X_{i},X_{j},X_{k}\in \left\{X,Y,Z\right\},$ the BROJA decomposition of $\left(T,\left(X_{i},X_{j}\right),X_{k}\right)$ is:

$$\begin{array}{cc} \tilde{\mathrm{CI}}\left(T;\left(X_{i},X_{j}\right),X_{k}\right) & =\delta \left(T;X_{i}X_{j}X_{k}\right)+\delta \left(T;X_{i}X_{k}\right)+\delta \left(T;X_{j}X_{k}\right)+\delta \left(T;X_{i}X_{k}.X_{j}X_{k}\right)\\ \tilde{\mathrm{UI}}\left(T;\left(X_{i},X_{j}\right)\setminus X_{k}\right) & =\delta \left(T;X_{i}\right)+\delta \left(T;X_{i}.X_{j}X_{k}\right)+\delta \left(T;X_{j}\right)+\delta \left(T;X_{j}.X_{i}X_{k}\right)+\delta \left(T;X_{i}.X_{j}\right)\\ \; & +\delta \left(T;X_{i}X_{j}\right)+\delta \left(T;X_{i}X_{j}.X_{i}X_{k}\right)+\delta \left(T;X_{i}X_{j}.X_{j}X_{k}\right)\\ \; & +\delta (T;X_{i}X_{j}.X_{i}X_{k}.X_{j}X_{k})\\ \tilde{\mathrm{UI}}\left(T;X_{k}\setminus X_{i},X_{j}\right) & =\delta (T;X_{k})\\ \tilde{\mathrm{SI}}\left(T;X_{i},X_{j},X_{k}\right) & =\delta \left(T;X_{k}.X_{i}X_{j}\right)+\delta \left(T;X_{i}.X_{k}\right)+\delta \left(T;X_{j}.X_{k}\right)+\delta \left(T;X_{i}.X_{j}.X_{k}\right). \end{array}$$

### Appendix D.2. Two Double Source Subsystems

These subsystems have the form $\left(T,\left(X_{i},X_{j}\right),\left(X_{i},X_{k}\right)\right)$ where $X_{i},X_{j},X_{k}\in \left\{X,Y,Z\right\},$$X_{i}\neq X_{j},$$X_{i}\neq X_{k},$ and $X_{j}\neq X_{k}$. Now, apply the BROJA decomposition to the subsystem (T,(X,Y),(X,Z)). Therefore, its four PID quantities are defined as follows:

$$\begin{array}{cc} \tilde{\mathrm{CI}}\left(T;X,Y\right)\; & =\mathrm{MI}\left(T;X,Y,Z\right)-\min_{Q\in \Delta_{P}^{XY.XZ}}\mathrm{MI}\left(T;X,Y,Z\right)\\ \tilde{\mathrm{UI}}\left(T;\left(X,Y\right)\setminus \left(X,Z\right)\right)\; & =\min_{Q\in \Delta_{P}^{XY.XZ}}\mathrm{MI}\left(T;X,Y,Z\right)-\min_{Q\in \Delta_{P}^{XY.XZ}}\mathrm{MI}\left(T;X,Z\right)\\ \tilde{\mathrm{UI}}\left(T;\left(X,Z\right)\setminus \left(X,Y\right)\right)\; & =\min_{Q\in \Delta_{P}^{XY.XZ}}\mathrm{MI}\left(T;X,Y,Z\right)-\min_{Q\in \Delta_{P}^{XY.XZ}}\mathrm{MI}\left(T;X,Y\right)\\ \tilde{\mathrm{SI}}\left(T;\left(X,Y\right),\left(X,Z\right)\right)\; & =\min_{\begin{array}{c} Q\in \Delta_{P}^{XY.XZ},\\ \mathrm{CoI}(T;X,Y;X,Z)=0 \end{array}}\mathrm{MI}\left(T;X,Y,Z\right)-\min_{Q\in \Delta_{P}^{XY.XZ}}\mathrm{MI}\left(T;X,Y,Z\right). \end{array}$$

Note that the (X,Y) and (X,Z) marginal distributions are fixed. Then, $\mathrm{MI}(T;X_{1},X_{2}),$$\mathrm{MI}(T;X_{1}|X_{2}),$$\mathrm{CoI}(T;X_{1};X_{2}),$ and MI(T;X:Y,X:Z) are invariant over $\Delta_{P}^{XY.XZ}$ for $\left(X_{1},X_{2}\right)=\left(X,Y\right)$ and $\left(X_{1},X_{2}\right)=\left(X,Z\right)$. Therefore, for $\left(X_{1},X_{2},X_{3}\right)=\left(X,Y,Z\right)$ and $\left(X_{1},X_{2},X_{3}\right)=\left(X,Z,Y\right)$, $\delta \left(T,X_{1}X_{2}\right)+\delta \left(T,X_{1}X_{2}.X_{1}X_{3}\right)+\delta \left(T,X_{1}X_{2}.X_{2},X_{3}\right)+\delta \left(T,X_{1}X_{2}.X_{1}X_{3}.X_{2}X_{3}\right)+\delta \left(T,X_{3}.X_{1}X_{2}\right)$ are fixed. However, from Assumption A2 and the two fixed (X,Y) and (X,Z) marginals, then the redundancies δ(T;Z.XY) and δ(T;Y.XZ) are invariant over $\Delta_{P}^{XY.XZ}$. Therefore, in addition to 2 in Assumption A2, the following partial information measures are invariant $\Delta_{P}^{XY.XZ}$:

1. $\delta (T;X_{i}.XX_{j}),$ for all $X_{i},X_{j}\in \left\{Y,Z\right\}$ since the $\left(X,X_{j}\right)$ marginal is fixed.
2. δ(T;XY.XZ)+δ(T,XY.XZ.YZ) since MI(T;X:Y,X:Z) and δ(T;Z.XY) are invariant.
3. $\delta (T;X_{i}),$ for all $X_{i},X_{j}\in \left\{Y,Z\right\}$ since $\mathrm{MI}(T;X_{i})$ and $\delta (T;X_{i}.XX_{j})$ are invariant over $\Delta_{P}^{XY.XZ}.$
4. $\delta \left(T,XX_{i}\right)+\delta \left(T,XX_{i}.X_{i}X_{j}\right),$ is invariant for all $X_{i},X_{j}\in \left\{Y,Z\right\}$ since $\delta (T;XX_{i}.XX_{j})$ + $\delta (T,XX_{i}.XX_{j}.X_{i}X_{j}),$ and $\mathrm{CoI}(T;X;X_{i}),$$\delta (T;X_{j}.XX_{i})$ are invariant over $\Delta_{P}^{XY.XZ}.$

Thus, using Assumption A2 and the definition of MI(T;X,Y,Z) over the redundancy lattice,

$$\begin{array}{cc} \min_{Q\in \Delta_{P}^{XY.XZ}}\mathrm{MI}\left(T;X,Y,Z\right) & =\delta (T,XY)+\delta (T,XZ)+\delta (T,XY.XZ)+\delta (T,XY.YZ)\\ \; & +\delta (T,XZ.YZ)+\delta (T,XY.XZ.YZ)+\delta (T,X)+\delta (T,Y)\\ \; & +\delta (T,Z)+\delta (T,X.YZ)+\delta (T,Y.XZ)+\delta (T,Z.XY)\\ \; & +\delta (T,X.Y)+\delta (T,X.Z)+\delta (T,Y.Z)+\delta (T,X.Y.Z). \end{array}$$

The synergy is evaluated as:

$$\begin{array}{cc} \tilde{\mathrm{CI}}\left(T;X,Y\right) & =\mathrm{MI}\left(T;X,Y,Z\right)-\min_{Q\in \Delta_{P}^{XY.XZ}}\mathrm{MI}\left(T;X,Y,Z\right)\\ \; & =\delta (T,XYZ)+\delta (T,YZ). \end{array}$$

The unique information of (X,Y) is expressed as:

$$\begin{array}{cc} \tilde{\mathrm{UI}}\left(T;X,Y\setminus X,Z\right) & =\min_{Q\in \Delta_{P}^{XY.XZ}}\mathrm{MI}\left(T;X,Y,Z\right)-\min_{Q\in \Delta_{P}^{XY.XZ}}\mathrm{MI}\left(T;X,Z\right)\\ \; & =\delta (T,XY)+\delta (T,XY.YZ)+\delta (T,Y). \end{array}$$

The unique information of (X,Z) is written as:

$$\begin{array}{cc} \tilde{\mathrm{UI}}\left(T;X,Z\setminus X,Y\right) & =\min_{Q\in \Delta_{P}^{XY.XZ}}\mathrm{MI}\left(T;X,Y,Z\right)-\min_{Q\in \Delta_{P}^{XY.XZ}}\mathrm{MI}\left(T;X,Y\right)\\ \; & =\delta (T,YZ)+\delta (T,XZ.YZ)+\delta (T,Z). \end{array}$$

When CoI(T;X,Y;X,Z)=0, then:

$$\begin{array}{cc} \delta (T,XYZ)+\delta (T,YZ) & =\delta (T,XY.XZ)+\delta (T,XY.XZ.YZ)\\ \; & +\delta (T,X)+\delta (T,X.YZ)+\delta (T,Y.XZ)+\delta (T,Z.XY)\\ \; & +\delta (T,X.Y)+\delta (T,X.Z)+\delta (T,Y.Z)+\delta (T,X.Y.Z). \end{array}$$

The shared information of X,Y and X,Z is evaluated as:

$$\begin{array}{cc} \tilde{\mathrm{SI}}\left(T;X,Y,Z\right) & =\min_{\begin{array}{c} Q\in \Delta_{P}^{XY.XZ},\\ \mathrm{CoI}(T;X,Y;X,Z)=0 \end{array}}\mathrm{MI}\left(T;X,Y,Z\right)-\min_{Q\in \Delta_{P}^{XY.XZ}}\mathrm{MI}\left(T;X,Y,Z\right)\\ \; & =\delta (T,XY.XZ)+\delta (T,XY.XZ.YZ)\\ \; & +\delta (T,X)+\delta (T,X.YZ)+\delta (T,Y.XZ)+\delta (T,Z.XY)\\ \; & +\delta (T,X.Y)+\delta (T,X.Z)+\delta (T,Y.Z)+\delta (T,X.Y.Z). \end{array}$$

Hence, then the BROJA decomposition of the subsystem $\left(T,\left(X_{i},X_{j}\right),\left(X_{i},X_{k}\right)\right)$ is:

$$\begin{array}{cc} \tilde{\mathrm{CI}}\left(T;\left(X_{i},X_{j}\right),\left(X_{i},X_{k}\right)\right) & =\delta \left(T;X_{i}X_{j}X_{k}\right)+\delta \left(T;X_{j}X_{k}\right),\\ \tilde{\mathrm{UI}}\left(T;\left(X_{i},X_{j}\right)\setminus \left(X_{i},X_{k}\right)\right) & =\delta \left(T;X_{i}X_{j}\right)+\delta \left(T;X_{i}X_{j}.X_{j}X_{k}\right)+\delta \left(T;X_{j}\right),\\ \tilde{\mathrm{UI}}\left(T;\left(X_{i},X_{j}\right)\setminus \left(X_{i},X_{k}\right)\right) & =\delta \left(T;X_{i}X_{k}\right)+\delta \left(T;X_{i}X_{k}.X_{j}X_{k}\right)+\delta \left(T;X_{k}\right),\\ \tilde{\mathrm{SI}}\left(T;\left(X_{i},X_{j}\right),\left(X_{i},X_{k}\right)\right) & =\delta \left(T;X_{i}X_{j}.X_{i}X_{k}\right)+\delta \left(T;X_{i}\right)+\delta \left(T;X_{i}X_{j}.X_{i}X_{k}.Y_{j}X_{k}\right)\\ \; & +\delta \left(T;X_{i}.X_{j}X_{k}\right)+\delta \left(T;X_{j}.X_{i}X_{k}\right)+\delta \left(T;X_{k}.X_{i}X_{j}\right)\\ \; & +\delta \left(T;X_{i}.X_{j}\right)+\delta \left(T;X_{i}.X_{k}\right)+\delta \left(T;X_{j}.X_{k}\right)+\delta \left(T;X_{i}.X_{j}.X_{k}\right). \end{array}$$

### Appendix D.3. Synergy of Three Double Source Systems

Consider the system of the form (T,(X,Y),(X,Z),(Y,Z)). The sources here are called composite, as they are compositions of the primary sources X,Y and Z. Applying the PID measure [38] based on maximum entropy decomposition (A13a–d) captures the synergy of composite sources only and cannot capture other contributions such as those involving unique or redundant composite sources; meaning that the optimization (A13a) is the only useful one for a system of composite sources. Therefore, using (A13a), the optimization is taken over the polytope:

(A25) $$\begin{array}{c} \Delta_{P}^{XY.XZ.YZ}=\left\{Q\in \Delta ;Q\left(T,X_{i},X_{j}\right)=P\left(T,X_{i},X_{j}\right)\;\mathrm{for}\;\mathrm{all}\;X_{i},X_{j}\in \left\{X,Y,Z\right\}\right\}. \end{array}$$

In this polytope, $\mathrm{MI}\left(X_{i},X_{j}\right),\mathrm{CoI}\left(X_{i},X_{j}\right),$ and $\mathrm{MI}(X_{i}|X_{j})$ are invariant for all $X_{i},X_{j}\in \left\{X,Y,Z\right\}$. Therefore, in addition to Assumption 2, the following partial information measures are invariant $\Delta_{P}^{XY.XZ.YZ}$.

1. $\delta (T;X_{k}.X_{i}X_{j}),$ for all $X_{i},X_{j},X_{k}\in \left\{X,Y,Z\right\}$ since the $\left(X_{i},X_{j}\right)$ marginal is fixed.
2. $\delta (T;X_{i}X_{j}.X_{i}X_{k}),$ for all $X_{i},X_{j},X_{k}\in \left\{X,Y,Z\right\}$ since $(X_{i},X_{j}),$$(X_{i},X_{k}),$ and $\left(X_{j},X_{k}\right)$ marginals are fixed.
3. δ(T;XY.XZ.YZ) since the (X,Y),(X,Z), and (Y,Z) marginals are fixed.
4. $\delta (T;X_{i}),$ for all $X_{i},X_{j},X_{k}\in \left\{X,Y,Z\right\}$ since $\mathrm{MI}(T;X_{i})$ and $\delta (T;X_{i}.X_{j}X_{k})$ are invariant over $\Delta_{P}^{XY.XZ.YZ}.$
5. $\delta (T,X_{i}X_{j}),$ for all $X_{i},X_{j},X_{k}\in \left\{X,Y,Z\right\}$ since $\mathrm{CoI}(T;X_{i};X_{j})$, $\delta (T,X_{i}X_{j}.X_{i}X_{k})$, $\delta (T,X_{i}X_{j}.X_{j}X_{k})$, $\delta (T,X_{i}X_{j}.X_{i}X_{k}.X_{j}X_{k})$, and $\Delta (T;X_{k}.X_{i}X_{j})$ are invariant over $\Delta_{P}^{XY.XZ.YZ}.$

Hence, the only partial information measure that is not fixed is δ(T;XYZ) and:

(A26) $$\begin{array}{cc} \min_{Q\in \Delta_{P}^{XY.XZ.YZ}}\mathrm{MI}\left(T;X,Y,Z\right) & =\delta (T,XY)+\delta (T,XZ)+\delta (T,YZ)+\delta (T,XY.XZ)\\ \; & +\delta (T,XY.YZ)+\delta (T,XZ.YZ)+\delta (T,XY.XZ.YZ)\\ \; & +\delta (T,X)+\delta (T,Y)+\delta (T,Z)+\delta (T,X.YZ)+\delta (T,Y.XZ)\\ \; & +\delta (T,Z.XY)+\delta (T,X.Y)+\delta (T,X.Z)+\delta (T,Y.Z)\\ \; & +\delta (T,X.Y.Z). \end{array}$$

The synergy is evaluated as:

$$\begin{array}{cc} \tilde{\mathrm{CI}}\left(T;\left(X,Y\right),\left(X,Z\right),\left(Y,Z\right)\right) & =\mathrm{MI}\left(T;X,Y,Z\right)-\min_{Q\in \Delta_{P}^{XY.XZ.YZ}}\mathrm{MI}\left(T;X,Y,X\right)=\delta \left(T,XYZ\right). \end{array}$$

### Appendix D.4. Computing the Finest Parts of the Trivariate PID

The values of δ(T;X),δ(T;Y),δ(T;Z),δ(T;X.YZ),δ(T;Y.XZ), and δ(T;Z.XY) are recovered from $\tilde{\mathrm{UI}}\left(X_{k}\setminus X_{i},X_{j}\right)$ of $\left(T,\left(X_{i},X_{j}\right),X_{k}\right)$ and $\tilde{\mathrm{UI}}\left(X_{k}\setminus X_{i},X_{j}\right)$ of (T,X,Y,Z), for all $X_{i},X_{j},X_{k}\in \left\{X,Y,Z\right\}.$

To recover the individual synergistic quantities, construct the following system of equations from the synergy of (T,X,Y,Z), (T,(X,Y),(X,Z),(Y,Z)), $\left(T,\left(X_{i},X_{j}\right),X_{k}\right)$, and $\left(T,\left(X_{i},X_{j}\right),\left(X_{i},X_{k}\right)\right)$ for all $X_{i},X_{j},X_{k}\in \left\{X,Y,Z\right\}.$

$$\begin{array}{cc} \tilde{\mathrm{CI}}\left(T;X,Y,Z\right) & =\delta (T;XYZ)+\delta (T;XY.XZ.YZ)+\delta (T;XY)+\delta (T;XZ)\\ \; & +\delta (T;YZ)+\delta (T;XY.XZ)+\delta (T;XY.YZ)+\delta (T;XZ.YZ)\\ \tilde{\mathrm{CI}}\left(T;\left(X,Y\right),Z\right) & =\delta (T;XYZ)+\delta (T;XZ)+\delta (T;YZ)+\delta (T;XZ.YZ)\\ \tilde{\mathrm{CI}}\left(T;\left(X,Z\right),Y\right) & =\delta (T;XYZ)+\delta (T;XY)+\delta (T;YZ)+\delta (T;XY.YZ)\\ \tilde{\mathrm{CI}}\left(T;\left(Y,Z\right),X\right) & =\delta (T;XYZ)+\delta (T;XY)+\delta (T;XZ)+\delta (T;XY.XZ)\\ \tilde{\mathrm{CI}}\left(T;\left(X,Y\right),\left(X,Z\right)\right) & =\delta (T;XYZ)+\delta (T;YZ)\\ \tilde{\mathrm{CI}}\left(T;\left(X,Y\right),\left(Y,Z\right)\right) & =\delta (T;XYZ)+\delta (T;XZ)\\ \tilde{\mathrm{CI}}\left(T;\left(X,Z\right),\left(Y,Z\right)\right) & =\delta (T;XYZ)+\delta (T;XY)\\ \tilde{\mathrm{CI}}\left(T;\left(X,Y\right),\left(X,Z\right),\left(Y,Z\right)\right) & =\delta (T;XYZ). \end{array}$$

This hierarchy that is needed to compute the trivariate PID quantities is implemented in script file test_trivariate_finer_parts.py.
